# A Lightweight Framework for Perception Analysis Based on Multimodal Cognition-Aware Computing

**DOI:** 10.3389/fnins.2022.879348

**Published:** 2022-05-26

**Authors:** Xuesheng Qian, Yihong Qiao, Mianjie Wang, Xinyue Wang, Mengfan Chen, Weihui Dai

**Affiliations:** ^1^Institute of Systems Engineering and Collaborative Laboratory for Intelligent Science and Systems, Macau University of Science and Technology, Macao, China; ^2^School of Management, Fudan University, Shanghai, China; ^3^College of Business, City University of Hong Kong, Hong Kong, Hong Kong, SAR China; ^4^Shanghai Ineutech Technology Co., Ltd., Shanghai, China; ^5^College of Letters and Science, University of California, Berkeley, Berkeley, CA, United States; ^6^China Science IntelliCloud Technology Co., Ltd., Shanghai, China

**Keywords:** lightweight framework, multimodal cognition-aware computing, VUCA environment, construction site, non-laboratory

## Abstract

The VUCA environment challenged neuropsychological research conducted in conventional laboratories. Researchers expected to perform complex multimodal testing tasks in natural, open, and non-laboratory settings. However, for most neuropsychological scientists, the independent construction of a multimodal laboratory in a VUCA environment, such as a construction site, was a significant and comprehensive technological challenge. This study presents a generalized lightweight framework for perception analysis based on multimodal cognition-aware computing, which provided practical updated strategies and technological guidelines for neuromanagement and automation. A real-life test experiment on a construction site was provided to illustrate the feasibility and superiority of the method. The study aimed to fill a technology gap in the application of multimodal physiological and neuropsychological techniques in an open VUCA environment. Meanwhile, it enabled the researchers to improve their systematic technological capabilities and reduce the threshold and trial-and-error costs of experiments to conform to the new trend of VUCA.

## Introduction

Nowadays, sensor technology has been rapidly developing, expanding its applications in industry and other fields. In psychology and cognitive science, experimental techniques and methods, such as electroencephalography (EEG), eye-tracking, and multichannel physiological monitoring, have emerged due to the successful application of sensor technology, significantly improving the scientific understanding of human cognition and behaviors.

There are two emerging trends in the advancement of instructions. First, techniques and methods in the behavioral and cognitive domains have changed from invasive to non-invasive and from explicit to implicit measures. For example, non-contact methods are less intrusive and more flexible for subjects. A second trend is that experiments using a single instruction have become insufficient to satisfy the requirements for increasingly refined cognitive and sensory detection. Social and economic development has demanded a more comprehensive exploration of science and technology.

In response to these two trends, researchers have proposed a concept called cognition-aware computing (Zander and Kothe, 2011; Bulling and Zander, 2014). This is a systematic approach that acquires multidimensional data to analyze the cognitive states, such as behaviors, and brain activities of users. The approach has prominent advantages. Particularly, in the volatile, uncertain, complex, and ambiguous (VUCA) environment, cognition-aware computing reduces the deficiencies associated with conventional self-reported driven research in psychology. Thus, neuropsychological perspectives are better suited for research in a non-conventional open laboratory environment, such as construction engineering and engineering management. Neuropsychological perspective and cognition-aware computing present an increasing potential with the continuous diversification of sensor and information technologies.

Cognition-aware computing with multiple devices is disparate from direct measurements using a single device following operating instructions. In some cases, the physiological device can be compatible with the access of other channel devices through the transmission line. Nevertheless, the environment covered by the approach is limited, and it is difficult to deal with the more complex experimental scenario of VUCA.

In contrast, the high-availability method has a unique technical threshold and requires systematic support to achieve command synchronization, data acquisition, and data fusion different from those in conventional laboratory scenarios. Although many scholars have realized and attempted to incorporate multimodal perception in scientific research, such application is limited by the lack of adequate technical support. A large number of studies on this topic are related to the mode of independent measurement even if with multiple channels. We refers which to “pseudo-multimodal cognition-aware computing”. The ideal multimodal method requires the utilization of a variety of instruments and analysis of diverse data. Therefore, to obtain a holistic framework, multimodal instruments and data can be aggregated and collaborated, thus building “a whole framework” in a real sense.

To solve these problems, this study presents a multimodality acquisition method based on the practical technical obstacles encountered in multimodal neuroscience and biobehavioral experiments in a non-laboratory, naturalized VUCA environment. This is a lightweight framework that supports real-time data aggregation, analysis, and interaction, and provides a systematic technological guide to neuromanagement and automation for researchers.

The other sections of this paper are organized as follows. Section Literature surveys works related to perceptual analysis and cognitive-aware computing. Section MCAC Framework introduces the proposed lightweight multimodal cognition-aware computing framework. Section Practice in Construction Site presents a real-life test experiment on a construction site and its operation results, followed by conclusions in Section Conclusion.

## Literature

### Perceptual Analysis

Human perceptual ability is a complex and integrated cognitive activity. Many researchers have conducted perceptual analysis studies of human cognitive activity. The model of human perception can be represented according to the physiological and behavioral information in the human brain. The model demonstrates that the information regarding human perceptual performance is stored in a particular form of expression, and the structure built by this stored information is combined with certain expectations, resulting in a specific behavior (Ogiela, 2017). Thus, perception is closely linked to cognitive and decision-making processes.

The perceptual analysis is a methodological attempt to study human perception. Since the performance of perceptual behavior relates to many factors, a single research method cannot answer all the questions about the nature of perception. The perceptual analysis is a means of explaining this process or understanding perception and experience. Nowadays, an increasing number of scholars have obtained results using perceptual analysis based on multichannel participation (Wang et al., 2021).

### Cognitive-Aware Computing Approach

#### Single-Mode Cognition-Aware Computing Approach

There are many applications for single-mode cognition-aware computing. For example, it can be employed in any cognitive perception study that uses a single-mode instruction, such as neural observation instruments and IoT devices. In neuropsychology, the application of BCI and eye-tracker technologies is the most popular. Generally, these techniques and methods are adequate, and based on these tools, researchers can reveal human decision-making behaviors.

#### Cognition-Aware Computing With Multimodal Approach

In complex perceptual studies, single-mode cognition-aware computing is deficient in interpreting cognitive dimensions. Therefore, more technologies are incorporated into the cognitive and perceptual computing systems. For example, researchers combined eye movements with EEG in the latest study to reflect a more refined cognitive picture (Carter and Luke, 2020). In addition, complex problems in human–computer interaction can be examined more accurately based on the integration of multimodal data, such as clickstream, eye movement, EEG, and wristband data (Giannakos et al., 2019).

However, in general, the above-mentioned experiments were conducted in exchange for more dimensional cognitive data through an inefficient and demanding experimental process and experimental requirements. This constraint makes the results of these experiments difficult to measure in non-laboratory natural settings, especially in the VUCA environment, such as engineering, construction, and emergency management. Hence, conventional methods are difficult to replicate in the laboratory measurement environment, which challenges the applicability of the research conclusions.

### Technology of Lightweight Framework

The lightweight framework is a method opposite to the heavyweight framework. Although researchers have not reached a consensus on the definition of a lightweight framework, their target scenarios are very specific, such as fast migration and deployment (Crawl et al., 2016), and quick and easy adaptation to changes in the business strategy (Kirk and Tempero, 2012). All in all, it is often considered that a lightweight framework does not come with an invasive interface. Moreover, it is not dependent on the container and is easy to configure, deploy, and generalize, as well as it is quick to start. Therefore, easy deployment, migration, and application are advantages of lightweight frameworks when compared to the industrial heavyweight ones. To sustain this consensus, this study considers the lightweight framework as a set of independent service implementations that can meet the requirements of easy migration, easy deployment, and fast response in multiple environments with low complexity, which facilities generalization and flexibility in meeting the requirements of research in the VUCA environment, as mentioned before, by using aggregated and collaborated multimodal instruments and data.

## MCAC Framework

Currently, it is challenging for most neuropsychological researchers to be free to choose the instruments and independently build a holistic multimodal cognitive framework under the VUCA environment. The main problem is technical challenges. The specific difficulties are the following: commonly used PCs have limited computing and I/O throughput capacity, it is difficult to carry out data acquisition and fusion of various instruments, the starting schedules of sensors' acquisition times are different, and wireless networks often have unpredictable delay fluctuations in non-laboratory environments. The above-mentioned difficulties need a systematic solution, which is addressed by the proposed lightweight multimodal cognition-aware computing (MCAC) framework.

### Framework Features

#### Multi-Layer Structure

**Figure 1:**
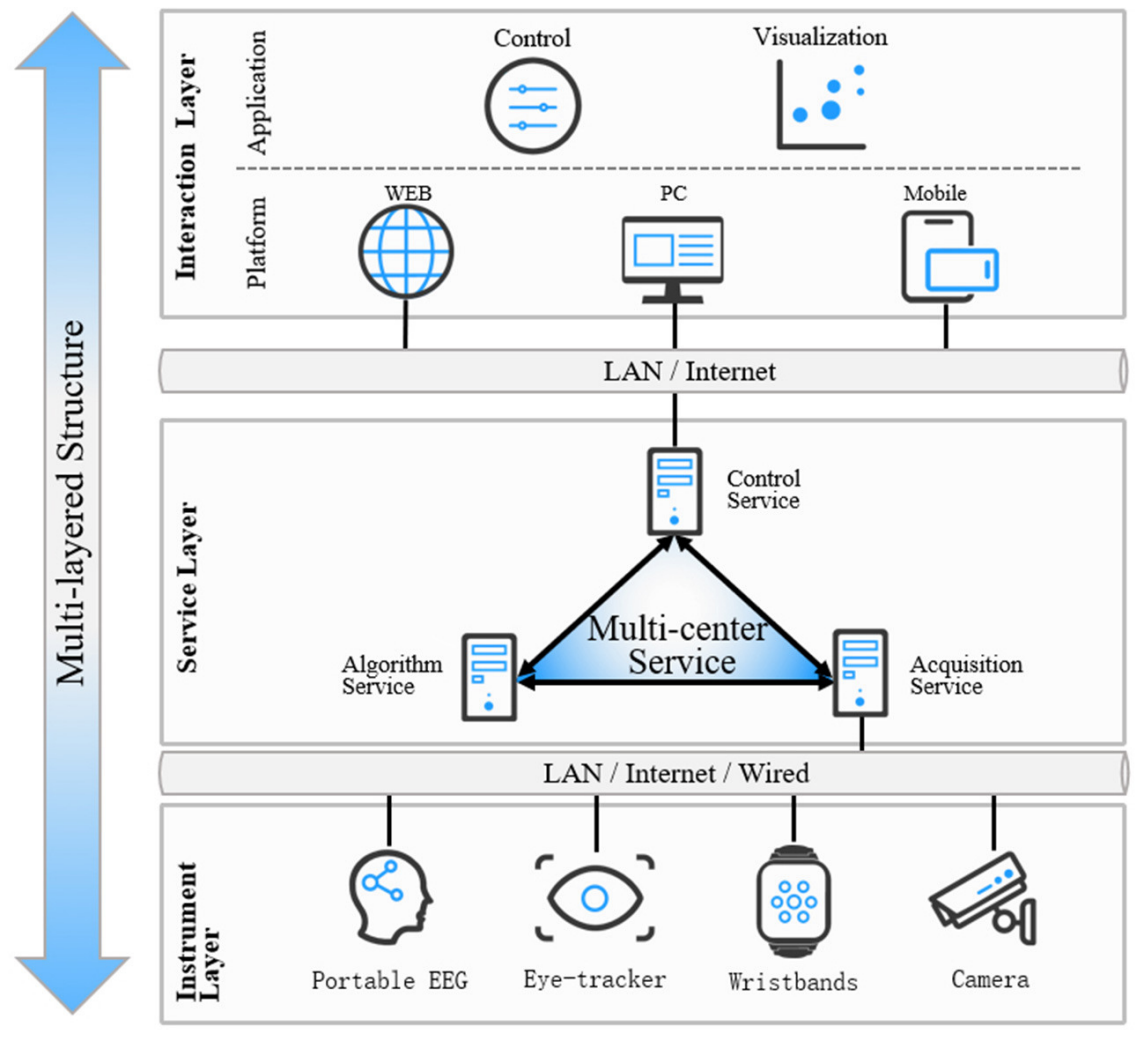
The MCAC framework architecture.

The MCAC framework described in this work is divided into three layers: instruments, service, and interaction (Figure 1).

The instrument layer includes instruments required for various experiments, such as EEG, eye trackers, and physiological instruments, which are usually applied in conventional unimodal cognition-aware computing systematic approaches. In particular, these instruments include both experimental instruments that are conventionally set up for experiments and equipment that are already available in open experimental scenarios (typically cameras). Incorporating these existing equipment in the study allows for the enrichment of data sources in the environment and reduces the additional costs related to the purchase of instruments, deployment, and dismantling. Therefore, if relevant instruments are already present in a particular environment, the possibility of including these instruments in future studies should be considered. All these instruments are connected *via* a LAN network or directly wired to the acquisition server. Internet connections are not recommended due to higher network latency with uncertainty, but can be chosen if necessary with time-delay corrections for data.

The service layer includes the acquisition, control, and algorithm services mentioned earlier, which is the main body of the whole framework and the primary focus of the study, and will be discussed in detail in the following sections.

The interaction layer includes application and visualization programs run on web, desktop, or mobile platforms. These applications can be connected to the service layer *via* a LAN or the Internet. The scalable form, which does not affect the main body of the service, supports various display forms and requirements, providing the possibility to transform the research scenario into practical applications, such as construction safety and smart retail.

#### Multi-Center Service

The core of the MCAC framework proposed in this paper is to adopt a loosely coupled approach with the separation of acquisition, algorithm, and control services at the service layer (Figure 1). The acquisition server obtains the required data directly from the sensors through the data interface or indirectly from the corresponding data port of the computer installed with the receiving software of the sensor. In addition, the acquisition server has extended sub-acquisition based on specific acquisition requirements and load conditions. The algorithm server supports real-time analysis of the collected data. The control server is used to receive instructions from the experimenter, control and coordinate the data and algorithm server, and function as process control. The acquisition, algorithm, and control servers are wired for connection to ensure reliability and deploy the same framework protocols.

In view of the capacity limitations of the PCs typically used in the neuropsychology experiment, the framework might be necessary to separate the acquisition service from the algorithm service, which effectively meets the high throughput requirements for the hardware to write high frequency (e.g., more than 512 Hz for a single EEG instrument) and return data in the acquisition server, provides computational resource required by the algorithm server, and ensures interaction commands to be processed with high priority. Thus, the three servers spread out different I/O throughput, computing resources, and network bandwidth pressure. Although the PC capacity is very limited compared to commercial servers, such a model is not likely to cause congestion in hardware.

Researchers can add or reduce corresponding parts according to their study needs. For example, in scenarios that do not require real-time analysis, the algorithm server can be omitted. In scenarios with the need for more sensor access or unique software installation, a parallel architecture with a group of acquisition servers is used to achieve the needs of large and complex experimental scenarios.

### Acquisition Service

**Figure 2:**
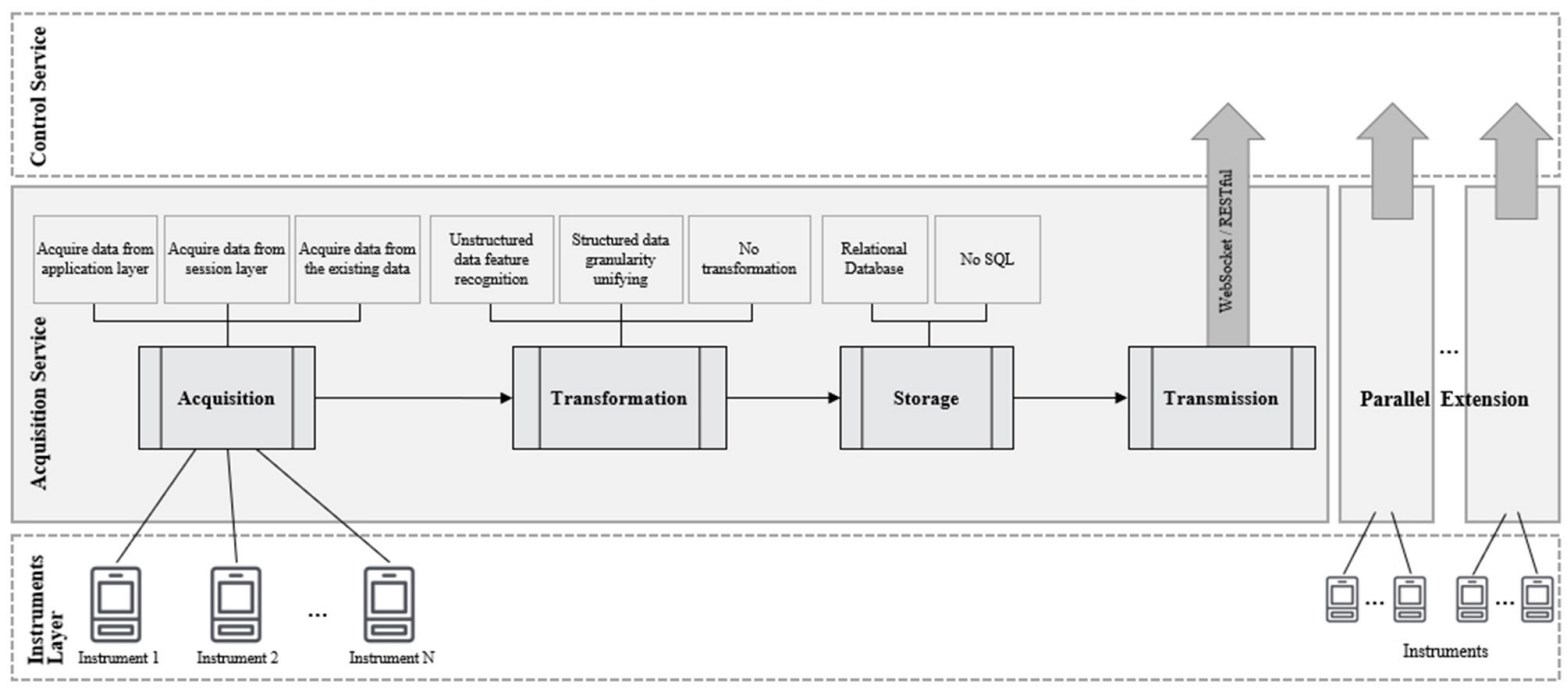
The flow of acquisition service.

The primary problem of the MCAC framework is how to acquire and preprocess the data collected from various sensors. The flow of the acquisition service is shown in Figure 2.

#### Original Data Acquisition

In neuropsychology, portable instruments, such as portable EEG, fNIRS, eye trackers, and portable physiological recorders, are mainly suitable for the VUCA environments rather than large laboratory apparatus, such as fMRI. Data acquisition methods are divided into three types:

- Acquire data from the application layer: this applies to acquiring data from the instruments in the application layer (referring to Open System Interconnection Reference Model, OSI) of the operation system directly, and it includes the devices that can customize the IP address of the data collector or be supported by the manufacturer with SDK (Software Development Kit) or API (Application Programming Interface). Representatives of such devices include ThinkGear AM (Neuro Sky, 2015), Tobii (Tobii, 2021), and TheEyeTribe (The EyeTribe, 2014). Also, Ramadan and Vasilakosc (Rabie et al., 2017) in their review listed eight kinds of popular BCI software platforms and tools most of which are open source. Based on the above, all can be achieved through simple programming data communication.
- Acquire data from the session layer: this applies to acquiring data from the communication port of the operation system in the session layer. Instead of directly obtaining data from the reserved interface, the method acquires data based on the programs running on the PC by listening to the data port of session communication. Such acquisition mode encompasses most equipment with closed software or streaming media, such as surveillance cameras and microphones. For audio and video streaming media systems, data can be resolved directly according to the common coding format, such as h.264/h.265. For the data from the special acquisition instrument of special structure, the instrument manufacturers can generally provide document support. Data reverse engineering based on instrument self-test is usually an effective process. It should be noted that the reverse engineering of data decoding should comply with the requirements of local laws, operation manuals of the equipment, and intellectual property protection guidelines, and one has to ensure that it is not used for commercial purposes.
- Acquire data from the existing data archive: this applies to acquiring data from two situations. First, most of the data can only be collected offline by using some special instruments. Second, some of the data itself is completed and has been collected, such as demographic information and neuropsychological test data collected offline or in advance. In this way, the existing or offline data can be integrated into the dynamic analysis with neural signal and behavioral data in a real-time setting.

#### Original Data Transformation

When multiple groups of independent instruments are used to collect the perceptual information of subjects, the data cannot simply be compiled together. One problem is that the sampling rates of different instruments are usually different. Generally, the sampling rate of a computer recording human–computer interaction behavior is 100 Hz, the sampling rate of an EEG sensor is 512 Hz, and the sampling rate of eye movement is 60-−200 Hz, and may even go up to 800 Hz. Another problem is that the data segments from different instruments have different meanings and cannot be simply spliced together. A typical example is the fusion of unstructured audio/video stream data. The proposed MCAC framework can handle the above-mentioned challenges by employing three data transformation methods corresponding to different structures and sampling granularity of the collected data:

- Unstructured data feature recognition: processing and coding is a common method that can be applied to unstructured data, such as audio and video streams in the field of social science research. Typically, researchers have done this by watching videos or listening to recordings. With the advent of advanced artificial intelligence tools, extraction of structural features from these unstructured data has become possible, and these techniques can be further used in the MCAC framework. For example, Dlib (Suwarno and Kevin, 2020) can capture the facial features in the video in real time, OpenPose (Qiao et al., 2017) can analyze human actions in real time, and Meta-Updater (Dai et al., 2020) can track multiple targets in the video for a long time. Researchers have embraced these techniques as an adjunct to neuroscience and biobehavioral observations (Qian et al., 2022). Thus, the key information in the audio and video streams can be extracted from the stream and transformed into a new data structure, organized in time series.
- Unifying granularity framework: the consistency in sampling granularity of the structured data is the key to fusion computing. According to different expectations and analysis requirements of the study, the method of data replication and filling or setting defect value can be selected to align the low sampling rate data into high sampling rate data, or averaging and moving window averaging technique can be used to reduce the frequency of high sampling rate data. It is worth noting that the performance load of the instruments is mainly concentrated in the I/O throughput of the acquisition server, so neither of the above two algorithms will have a greater impact on the performance of the acquisition server.
- No transformation is required: the data in the scenario are structured and have a consistent temporal sampling granularity. For example, if the subject's eye movement and active selection behaviors are collected simultaneously at the granularity of 512 Hz, the collected data can be directly spliced into a complete time series with a time scale of 0.01 s.

#### Original Data Storage

If storage conditions are met, all the original data should be stored locally at the acquisition server to ensure the traceability of all the experimental data in the future. Experiments requiring public natural scenes and industrialization scenes need to comply with local legal requirements for personal privacy protection.

In the proposed MCAC framework, different databases can be selected for storage. The relational database has the advantages of better operability and universality, which is suitable for the storage needs of most structured data. NoSQL databases can also be involved, particularly for the unstructured data or large-scale sparse data storage generated in a natural experiment or industrial scenes, which can effectively improve the storage and access efficiency.

#### Original Data Transmission

All data acquired will be transformed into a new data structure with a consistent model and consistent data granularity according to the established rules of the control service, and will be transmitted to the control service.

To balance convenience, reliability, and network efficiency, lightweight WebSocket or RESTful is the preferred way of data transmission. WebSocket is a concise and persistent communication protocol based on TCP connection, which realizes the full-duplex communication between the client and the server. It has the advantages of light data format, low-performance overhead, and efficient communication. It is one of the most popular network link methods for fast and efficient communication. RESTful is a design style and developmental method for resource location and resource operation of web applications based on HTTP protocol. It can be defined in XML format or JSON format for more concise, hierarchical, and easy cache operation. At present, RESTful is widely used in the interface technology of mobile Internet. The above two technologies can effectively solve the problems of link establishment, data access, long connection maintenance, packet loss retransmission, and so on in a standardized way in most experimental scenarios. They are very simple to implement, highly portable, and extensible, and hence meet the various requirements of an MCAC lightweight framework.

#### Parallel Acquisition Extension

The computing resources of a lightweight framework are limited. In this, the most commonly used equipment is a normal PC, which is essential for all neuropsychological labs. In the meantime, dozens of different types of experimental acquisition instruments may be accessed and long-term tests continuously carried out for a long time in the VUCA environment supported by the MCAC, and therefore, it is necessary to adopt a scalable parallel architecture.

**Figure 3:**
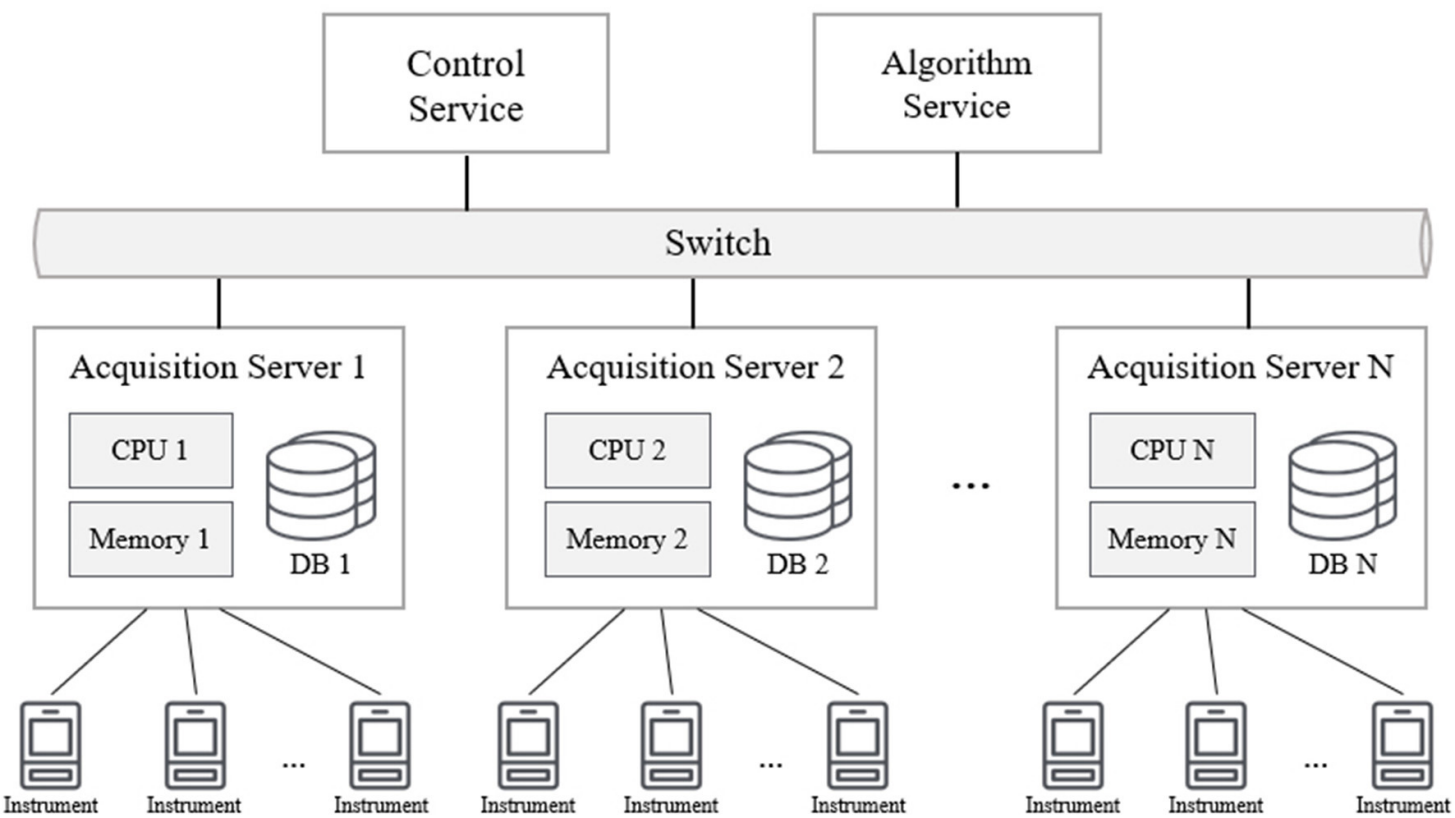
The parallel acquisition architecture.

Multiple clusters consisting of acquisition servers are connected with the control server to form a parallel storage architecture (parallel database) that is prevalent in the industry today. In the lightweight framework of MCAC, each acquisition server performs data extraction, transformation, and storage operations independently, making the parallel architecture a loosely coupled architecture for the shared data, as shown in Figure 3. Each database structure can be completely heterogeneous due to the different collection instruments that correspond to each collection server.

This structure has excellent scalability, and by only adding additional processing nodes (server), the processing capacity of the framework can be increased in a nearly linear proportion. Compared with the tightly coupled shared memory mode, the loosely coupled mode is more convenient to implement. Currently, main BMSs can support this parallelization extension easily.

It is worth noting that in the field of computer science, parallel database and distributed database are two different architectures, though they are both produced to improve database performance and availability. However, in the environment targeted by this study, the purpose of the parallel extension is to expand the processing capacity of the acquisition and meet special acquisition conditions. For example, to avoid run conflicts caused by some programs running at the same time. It is more important for social science researchers to know and apply this extension model so that all the acquisition instruments in the experiment become a whole system, rather than delving into the differences in the principles of the architecture.

### Control Service

**Figure 4:**
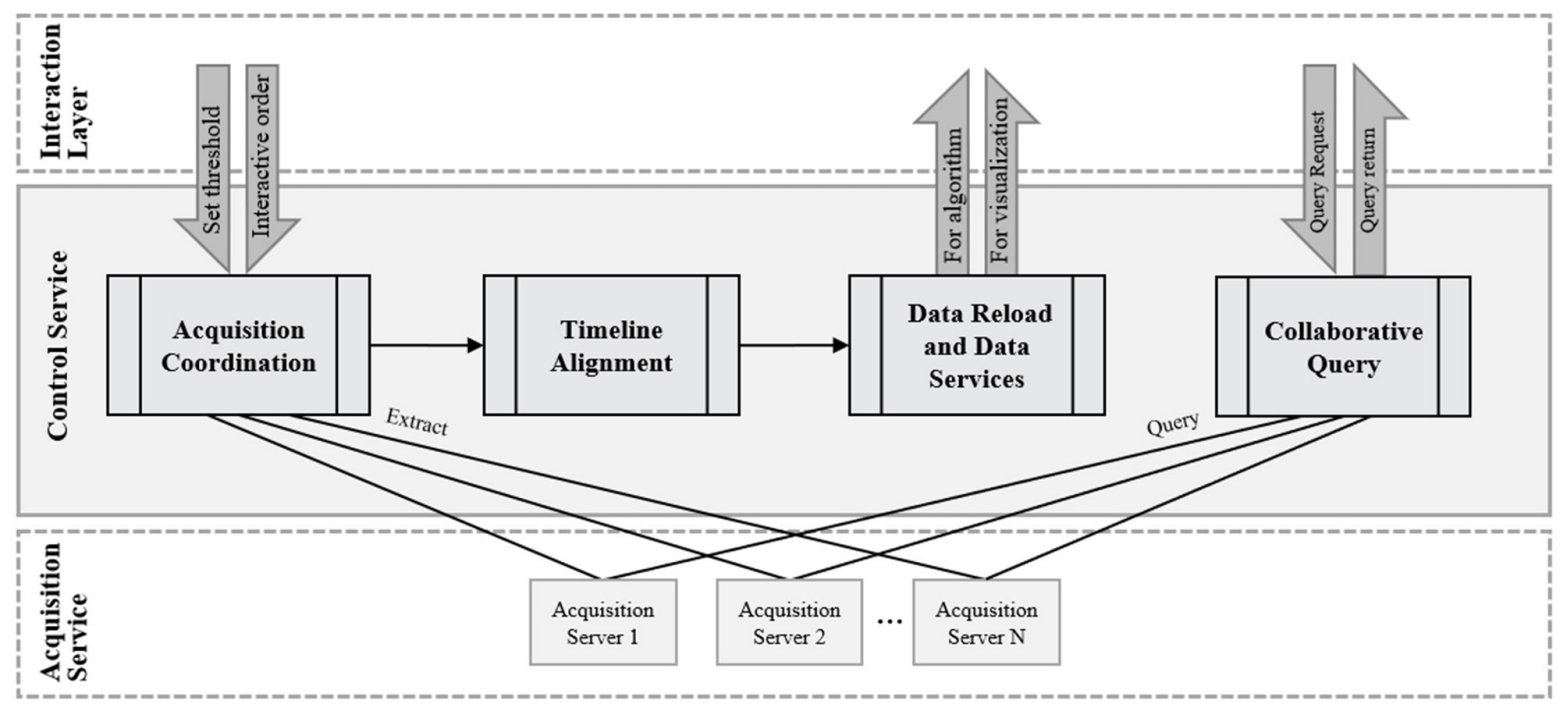
The flow of control service.

The control service of the MCAC deals with the alignment problem after data collection and provides data support for the subsequent analysis and interaction process. The flow of the control service is shown in Figure 4.

#### Acquisition Coordination

**Figure 5:**
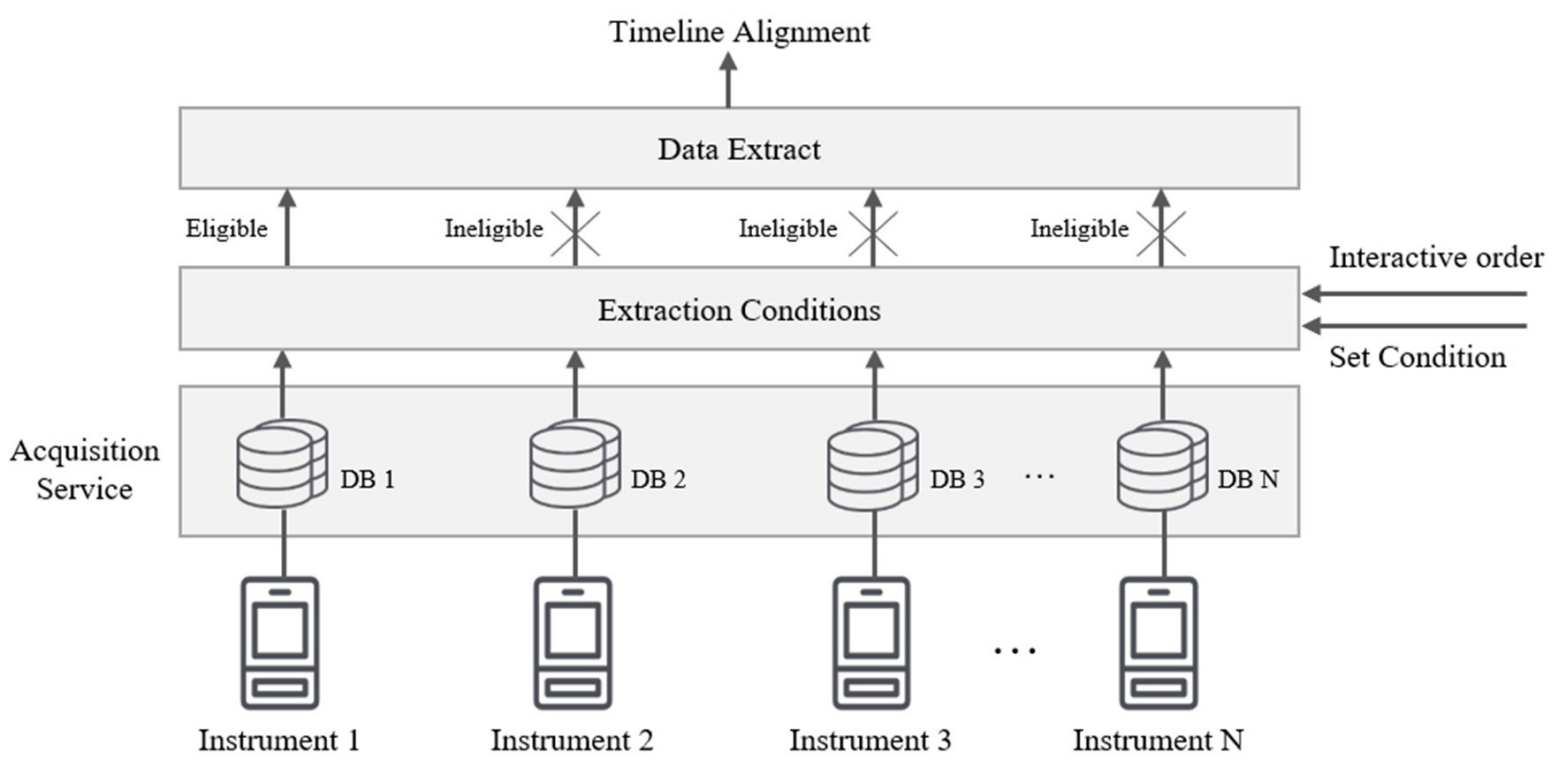
The flow of acquisition coordination.

Due to the complexity and openness of the VUCA environment, it may be difficult to ensure a straightforward and fixed test process like a conventional laboratory test. For example, subjects' information cannot be confirmed before the experiment, since all the experimental equipment cannot start simultaneously. Specifically, in the follow-up experiment examining the behavior of a construction safety inspector, the surveillance cameras at each point remain open. In this process, all personnel on the site are captured by the camera. However, the experiment will not begin until the safety inspector with a portable EEG is captured by a camera. Typically, this part of the work will require a lot of human effort to view and edit video clips, but in the proposed MCAC framework, this will be achieved by the automatic collaboration of the control service. The process of acquisition coordination is shown in Figure 5.

#### Timeline Alignment

In neuropsychological research, tiny inconsistent timelines may have a great impact on the final results. However, in the VUCA environment where the MCAC is deployed, some devices acquire data through wireless communication, causing inevitable time delays due to the physical network. Therefore, it is necessary for the control service to make each device align with the timeline when their data is combined.

**Figure 6:**
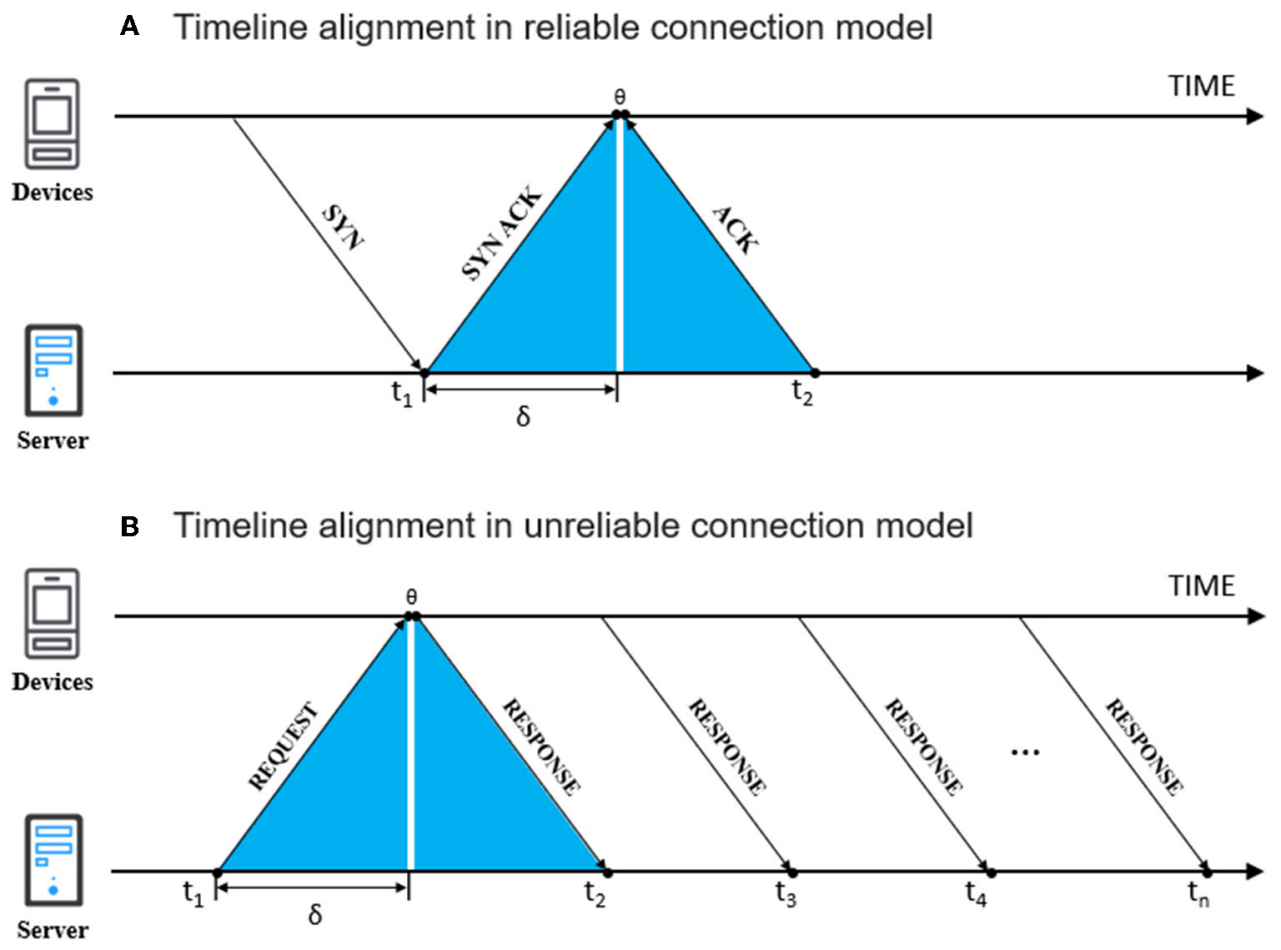
The timeline alignment strategy in two model.

In the field of computers, there are two main network time protocols for time alignment, NTP and PTP. On the other hand, since the wireless acquisition instruments used in the neuropsychological research are mainly IoT embedded devices that rarely support NTP or PTP services, the framework needs targeted adjustments. There are two modes (reliable connection and unreliable connection), and the timeline alignment strategies are shown in Figure 6.

Reliable connection mode (Figure 6A) is primarily for network interactions that require keeping a connection and is mainly represented by the TCP protocol. In this mode, the server and the acquisition device will be in continuous interaction during the transmission. We can consider the time that the server receives the client's first round of interaction instruction (SYN ACK) as t_1_ and the time the server receives the first data packet (ACK) as t_2_. Because the front-end acquisition device does not involve complex computation, the instrument processing time, theta (Θ), can be regarded as a constant approaching 0. Thus, the network transmission in-transit delay, delta (δ), can be calculated by (t_2_-t_1_)/2. In order to avoid the impact of network fluctuation, the process can be repeated for 10 times or more, and then calculate its trimmed mean.

Unreliable connection mode (Figure 6B) is primarily for network interactions that do not require keeping a connection and is mainly represented by the UDP protocol. In this mode, some servers and the acquisition device will not be in continuous interaction during the transmission after the transfer has started. Since the sensor is transmitting data back at a fixed acquisition frequency, the instrument processing time (Θ) can also be regarded as a constant approaching 0. Taking the response of the first 10 data packets or more, the network transmission in-transit delay is calculated as δ. In this situation, the δ value is equal to.


$$\begin{array}{l} TRIMMEAN\left\{\left(t_{n}-t_{1}\right)/\left(n-1\right)\right\} \end{array}$$

It is worth noting that the premise of the above calculation is that the in-transit delay of the experimental network transmission is symmetric. But due to network fluctuation and congestion, transmission in-transit delay is different even in the same wireless LAN. Therefore, the above-mentioned model is only a modification of the theoretical model. At the same time, researchers need to pay attention to the network stability of the acquisition instruments in the VUCA environment, and reduce the network instability through network separation and acquisition service expansion as far as possible. In this study, it is considered that it is not reliable that two times of delta (δ) is large than 0.8 times the derivative of the model's default frequency. For example, calculating the model default frequency of 10 Hz needs the transmission in-transmit delay <40 ms.

#### Data Reload and Data Services

**Figure 7:**
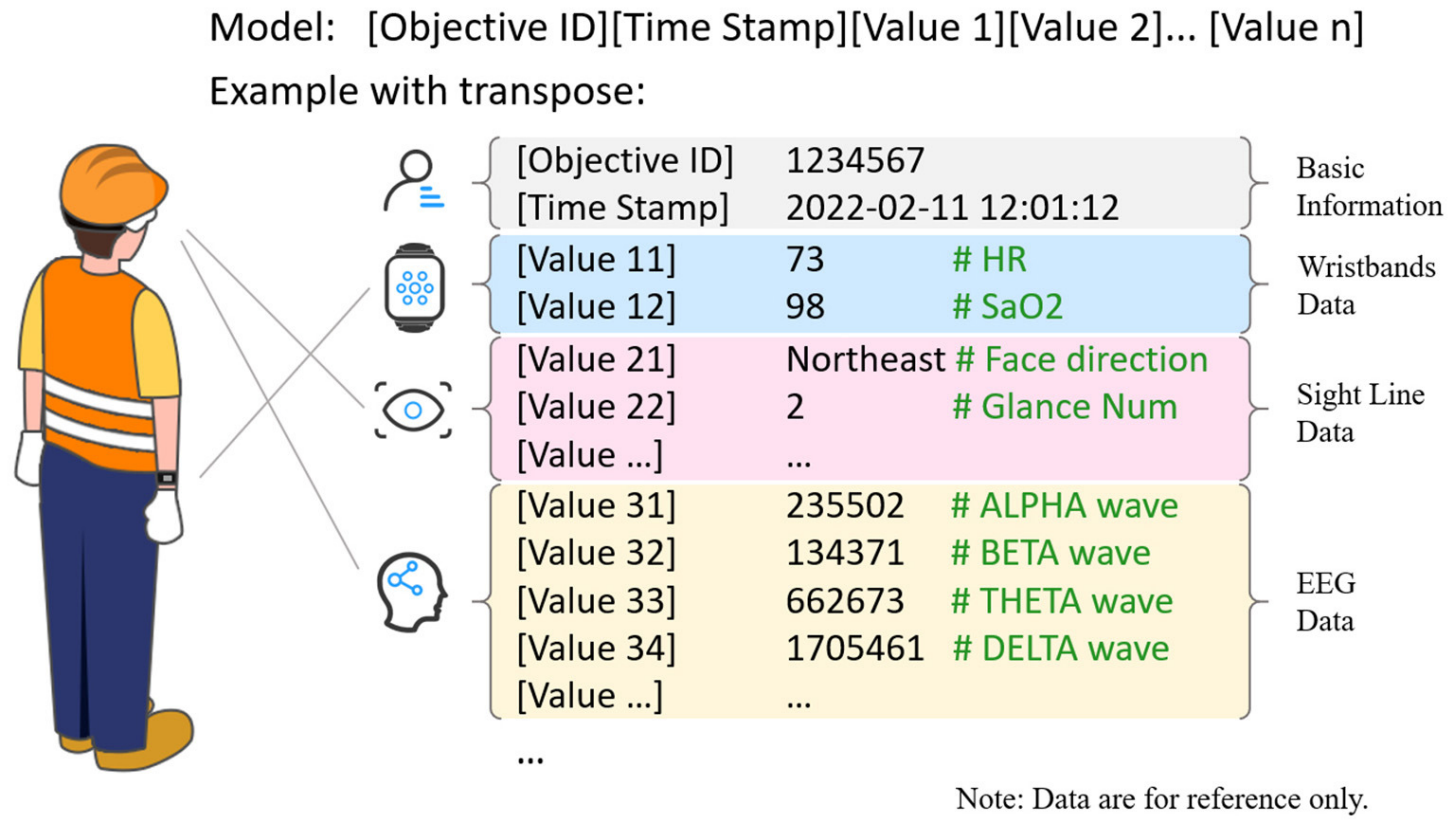
The recommendation model and example.

After acquisition coordination and timeline alignment, the control server will combine the data from different instruments to form the following recommendation model that one objective's all dimensions at each time point will be aggregated into one row, the model and example as Figure 7. Next, load them into the structured database of the control server.

The database provides data services for analysis and interaction servers. If specific analysis and display are necessary, the above data formats can also be optimized to improve the efficiency of real-time analysis and display.

#### Collaborative Query

**Figure 8:**
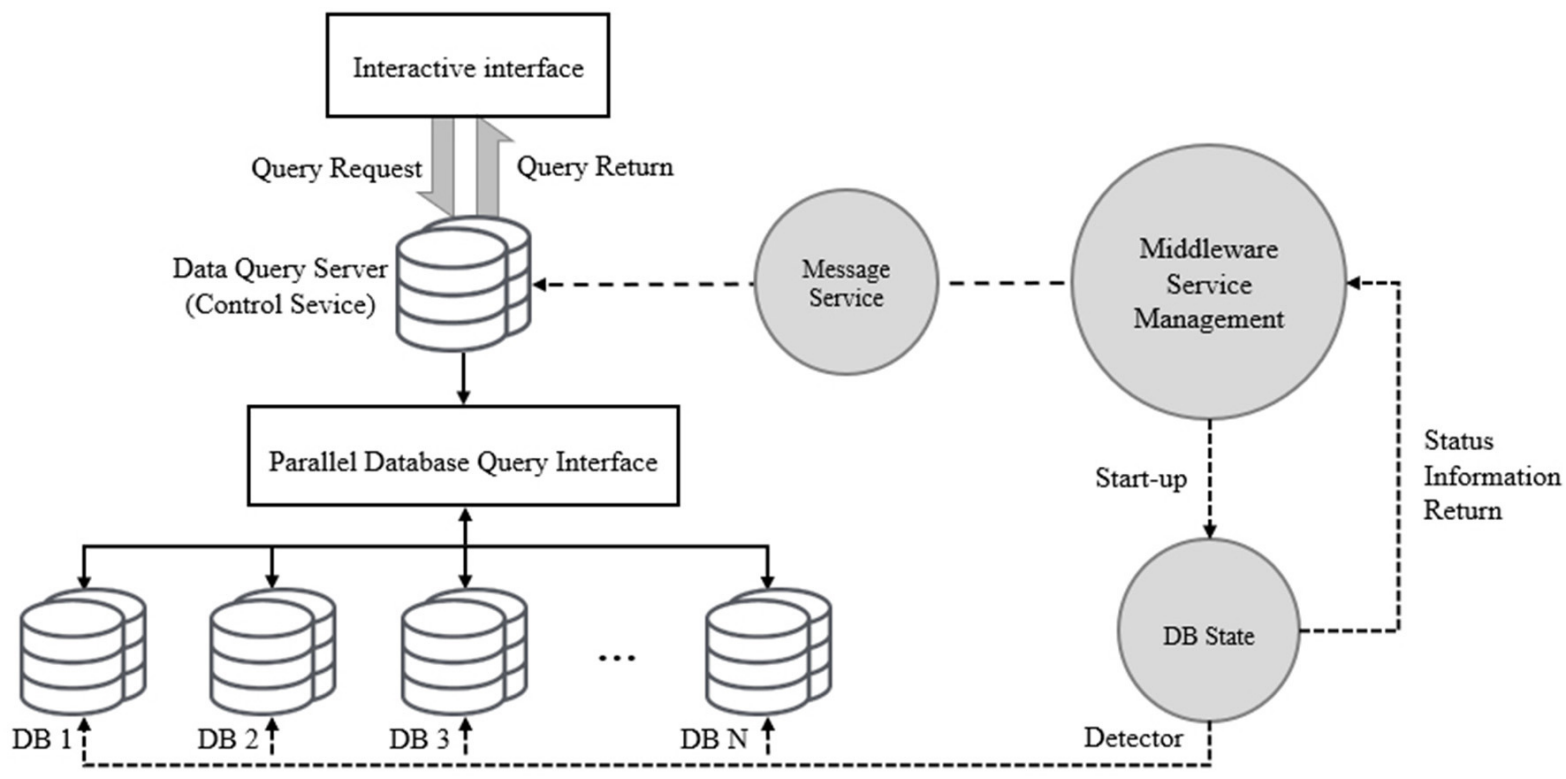
The flow of collaborative query.

The parallel extension service in MCAC should also be able to handle the subsequent query requirements for the original test stored in different acquisition servers. The queries for this requirement flow out are shown in Figure 8.

The figure shows that the interaction layer provides an interactive interface with the experimenter, accepts query requests for specific data, and returns query results. The control service centrally processes query requests in the data query server which deploys process logic and data dictionary, whether a parallel query or not.

Since the MCAC is not designed for industrial scenarios, it does not involve the complex development of industrial load balancing, failure transfer, data isolation, and low-level efficiency optimization. Also, the lightweight framework's basic query and loading needs can be met by packaging technologies, such as ODBC and JDBC.

Specifically, when a query service request is received from the control service, it will be sent to the query server which is located in the control server through the parallel database query interface. The query server generates the parallel query execution plan, and performs the query task flow in all parallel extended collection servers (database nodes) of the MCAC framework through the parallel data query interface, including control of parallel execution and data transfer between nodes. During this process, the query server is responsible for overall control and task synchronization. After the query is executed, the execution results of each node are summarized to the query server node and returned to the requester in the interaction layer by the control service. In order to ensure the reliability and correctness of the data query, the middleware Service Manager module constantly detects the execution process and returns it as a message to the control side (the data query server).

### Analysis Service

The core of analysis service is the integration of algorithms and automation. Typically, nonlinear analysis methods are commonly used in EEG data analysis and processing, but these methods are very much dependent on offline analysis after test completion. The MCAC that provides real-time preliminary results based on the consolidated data is logic. Multidimensional analysis is possible based on the preprocessing and alignment of multidimensional data at the acquisition server and control server in the MCAC framework. It is useful to be able to get preliminary analysis results in real time along with the testing process, which can provide more diverse possibilities for research, such as support for complex interactive research.

### Interaction Layer

The interaction layer of the MCAC is mainly presented in the form of a software system and undertakes the human–computer interaction with the whole framework. Because of the design pattern of the separation of data, service, presentation, and interaction in the MCAC framework, all the interactive applications of the web, desktop client, and mobile application, according to the needs of users and experiments, are available in MCAC in order to interact with users more efficiently under the lightweight framework.

Furthermore, relying on the structured data model at the control service, MCAC laid the groundwork for the use of almost any language and specification, including HTML, XHTML, CSS, flash, MathML, scalable vector graphics (SVG), Java, JavaScript, Adobe Flex, and other mainstream interaction modes.

It is important to note that the design pattern of MVC (Model-View-Controller) should be considered to maintain the high availability of a lightweight framework in a complex VUCA environment. The model layer undertakes the task of a business module, the view layer undertakes the interaction between view presentation and users, and the controller layer accepts the interaction request information of the view layer. By separating MVC's three-tier functions, the coupling degree of framework code is reduced. It is also conducive to the reuse of multiple components and supports the migration and extension of the framework.

## Practice in Construction Site

A construction site is a typical VUCA environment because of its cluttered and open environment, and complex and irregular material. The construction industry is universally accepted as one of the most dangerous industries. Therefore, many scholars have focused on a series of behavioral and decision-making studies in construction management.

This study illustrates the feasibility and superiority of the described MCAC framework for a specific research application in construction management. It provides technical guidelines for researchers to set up an experimental environment when conducting complex measurements with multimodal devices in a VUCA environment.

### Description

Safety inspection at construction sites has always been essential to ensure the safety and progress of the project. However, as a non-productive activity in real-life construction, it is easily neglected. Common problems have attracted much scholarly attention, including safety inspection not covering all areas, safety inspectors not being careful enough, and fatigue operations. Yet, previous technological limitations have led to studies conducted in simulated laboratory environments. Mobile brain imaging instruments (such as portable EEG and fNIRS) and wearable eye-tracking instruments have promoted the conductance of several studies (Li et al., 2019; Liao et al., 2021; Cheng et al., 2022). However, these artificially created “out- laboratories” are significantly different from the natural VUCA environment of a construction site, so the experimental results were questioned about the reproducibility of research and robustness of scientific findings.

### Methodology

For the purpose of this experiment, the ideal research method is to equip inspectors with neural sensing devices during a safety inspection and record their movement and behavior on-site simultaneously.

#### Conventional Method

Experimental procedure: first, researchers select a construction site for screening and stack some construction materials (items to be screened). Then the participant inspectors put on a neuro-observation instrument (to detect cognitive activity) and wearable eye-tracker (to detect line of sight). The researchers observe and record timestamps of each subject's behavior in detail and videotape the entire experimental process. For the operability of experiment, the subjects' routes and activities cannot be fully open instead of following certain experiment restrictions.

Data processing: The manual workload of the data acquisition stage in conventional methods is onerous and time-consuming. Valid EEG data and behavioral data are manually selected. The corresponding signal data from the overall test data of EEG and eye-tracking have to be segmented according to the timestamps of subjects' behaviors, while the audio and video signals have to be encoded for the target test subjects manually by the researchers.

Moreover, for several reasons, with the above-mentioned methods, the acquired data would be far away from the actual data in the natural and practical scenarios. First, considering the use of sensors, such as wearable eye-tracking instruments, subjects are imposed strong psychological hints on carefully facing each test, which can interfere with the results. Second, the compulsive construction safety rules of wearing helmets for all on-site personnel restrict the applicability of neural observation instruments. Third, the harsh environment of the construction site poses a risk to the sophisticated instruments. Finally, the testing paradigm requires staffing commitment and occupying construction sites for a long time, both of which hinder the involvement of subjects and raise the threshold for research.

In addition, even after overcoming the above difficulties, such a research method is merely “simulate-natural,” since the scenario cannot be reproduced in a daily real-site environment. In other words, the events that occurred can only be observed in an experimental study environment, which is not the original purpose of the research “outside the laboratory” environment and “into the VUCA environment”.

#### The MCAC Method

The VUCA environment needs data collection and studies based on large samples and regularity, which can be effectively supported by the MCAC framework proposed in this paper.

Experimental procedure: a portable EEG with fewer channels is used, and surveillance cameras deployed inside construction sites replace wearable eye-tracking instruments. The algorithm identifies the behavioral features of the subjects, such as action trajectories, movement, and direction of vision, from the video stream signal of the surveillance cameras. Based on the MCAC framework, subjects' behavioral features can be employed as trigger conditions to identify the EEG and behavioral features at each detection point automatically. Thus, the experiment can allow safety inspectors to conduct activity within the vast area of the construction site without restrictions.

It is worth noting that other workers may appear during the experiment or multiple subjects may be tested at the same time in the VUCA environment, which is also a situation that MCAC needs to deal with. The MCAC can mainly rely on the following two approaches: First, in cases of some devices binding with a specific subject, the device ID is naturally the unique identification. Second, the subjects could be distinguished by computer vision. It can be based on the identification of features worn by participants, such as the special color of a helmet and even the characteristic height and posture of participants. The continuous mobility of the subjects' real-time coordinates will be monitored by the algorithm to maintain the unique binding of ID and subject behavior until he/she leaves the detection area.

**Figure 9:**
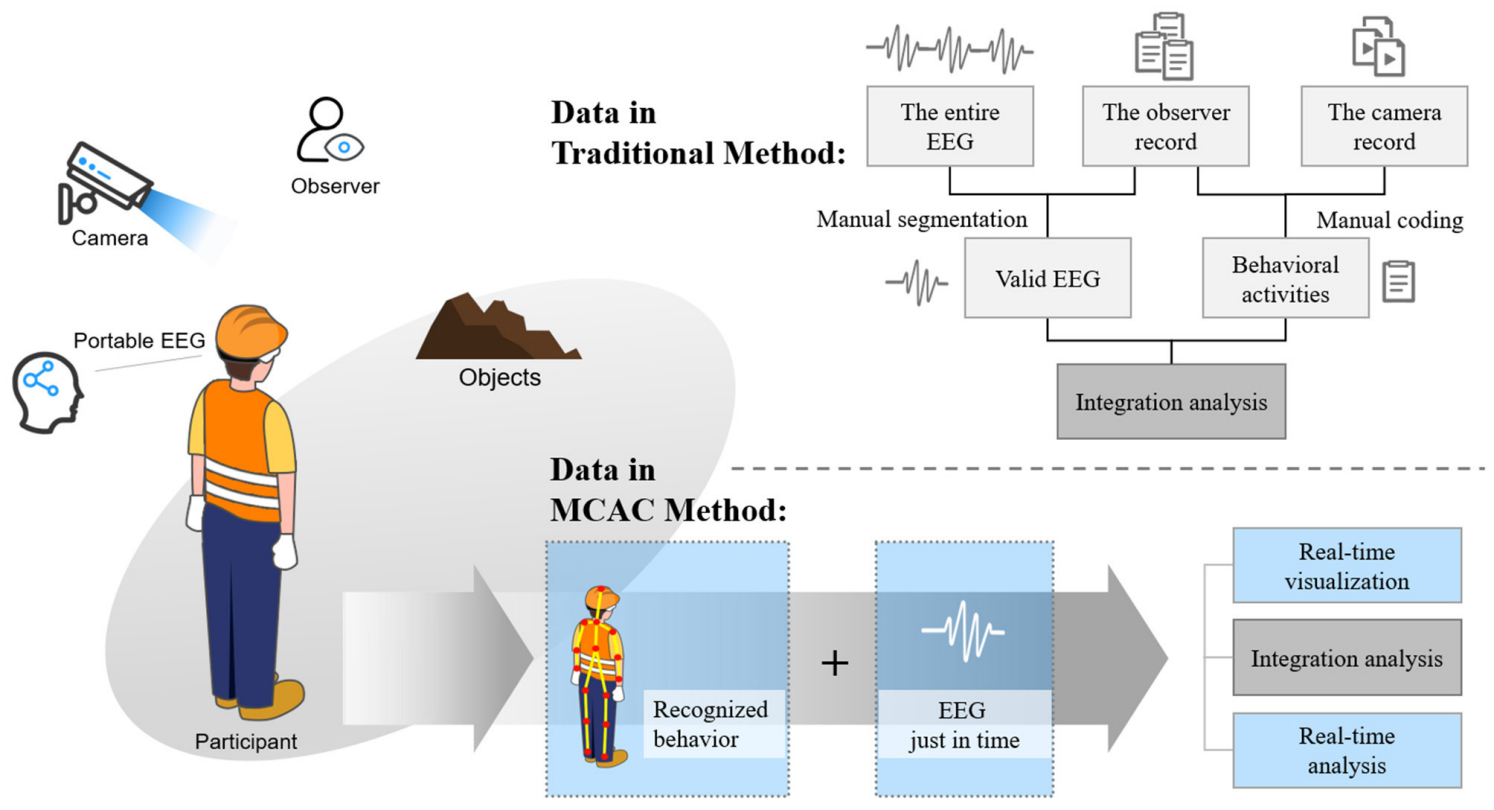
The comparison of MCAC and conventional method in the same scenario.

In this way, data of each subject are claimed for real-time analysis and feedback. The improvement simplifies the subsequent data analysis and makes the test scenario close to the natural environment. The comparison of this advantage is shown in Figure 9.

Based on the comparison shown in Figure 9, we can clearly see the advantages of the new technology framework proposed in the paper for multi-mode complex experiments in large-scale complex scenes. The behavioral and physiological data of the subjects can be immediately aggregated by the framework, and WYSIWYG analysis can be realized. Comprehensive technological support allows for more ambitious experimental programs that allow subjects to carry out more open-ended tasks in a larger space. Compared with the conventional methods that rely on manual data editing, data processing, and data merging, it is not only more convenient and efficient, but, in terms of methodology, it can also produce more original and abundance data to achieve the more enrichment of data discoveries by the most natural ways in data aggregation and automatic algorithm data analysis.

### Architecture Deployment

**Figure 10:**
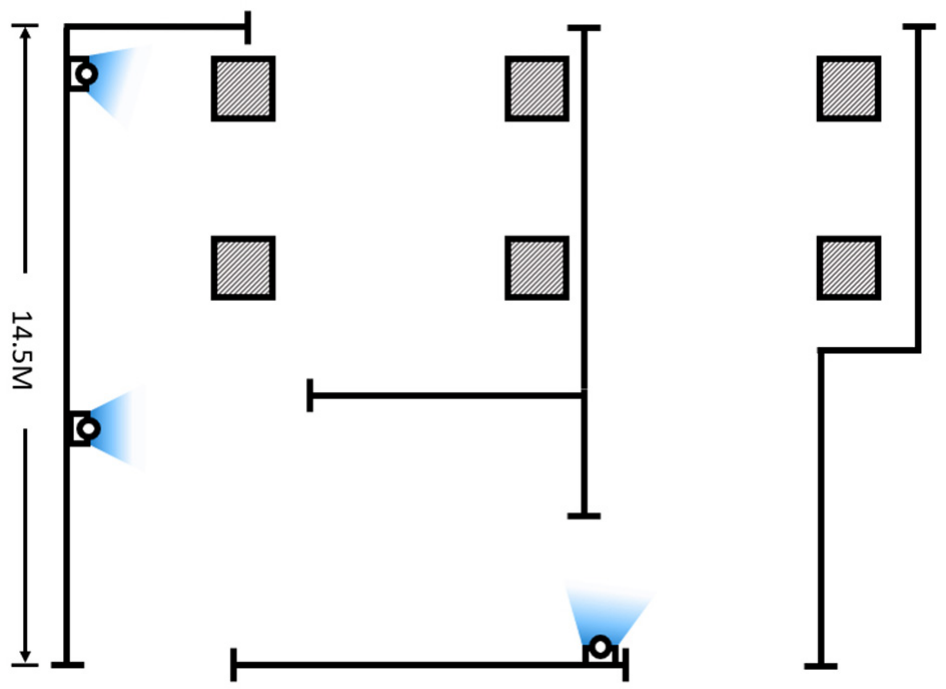
The diagrammatic sketch of experiment construction site and camera sets.

The strategy of the MCAC framework deployed in the experiment construction site is shown in Figure 10. There were three cameras covering more than 200 m^2^ of the construction site connected by wires, which allowed everyone including the safety inspector to move freely. There were three laptops that functioned as acquisition server, control server, and algorithm server. In addition, there was one portable EEG device that was developed based on ThinkGear AM and connected wirelessly by UDP protocol. The parallel extension was not needed.

The service module of the framework in the experiment is described earlier. The time granularity of the final analysis model is 100 ms (a moving window is used to reduce the granularity of EEG data).

The recognition algorithm in the experiment practice, the human skeleton point detection algorithm, and the face orientation detection algorithm adopt the commercial algorithm of intellicloud, but they can also be replaced by OpenPose, Openface, solvePnPRansac, or the combination of OpenCV, Dlib, and Numpy. All these algorithms can be accessed in the open-source algorithm library, and the technical parameters of equipment are presented in Table 1.

**Table 1:** Technical parameters of the selected camera.

**Parameter name**	**Parameter value**
Sensor type	1/1.8-inch CMOS
Pixel	4 million
Maximum resolution	2,688 × 1,520
Electronic shutter	1/3 s~1/100000 s
Wide dynamic	120 dB
Signal to noise ratio	>56 dB
Video compression standard	H.265; H.264; H.264H; H.264B; MJPEG
Using code stream	4,096 kbps (4M)
Optional video bit rate/KBps	H.264:32Kbps~10240KbpsH.265:12Kbps~10,240 Kbps
Video frame rate	50Hz: Primary code stream (2688 × 1520@25fps), Auxiliary code stream 1(704 × 576@25fps), Auxiliary code stream 2(1920 × 1080@25fps)
Access standard	ONVIF(Profile S/Profile G); GB/T28181;CGI;RTMP;

The technical parameters of the selected camera are listed in Table 1.

The technical parameters of the selected laptop are listed in Table 2.

**Table 2:** Technical parameters of the selected laptop.

**Parameter name**	**Parameter value**
Operating system	Windows 10
Processor	intel i5 10210u
Processor frequency	4.2 GHz
Graphics card	Integrated graphics card
Hard Disk	SSD-512 GB
Memory capacity	16 GB
Memory frequency	2,666 MHz
Memory type	DDR4

The technical parameters of the selected wireless network are listed in Table 3.

**Table 3:** Technical parameters of the selected wireless network.

**Parameter name**	**Parameter value**
Wireless operating frequency band	2.4 GHz
Wireless rate	5,400 Mbps

### Operation Results

**Figure 11:**
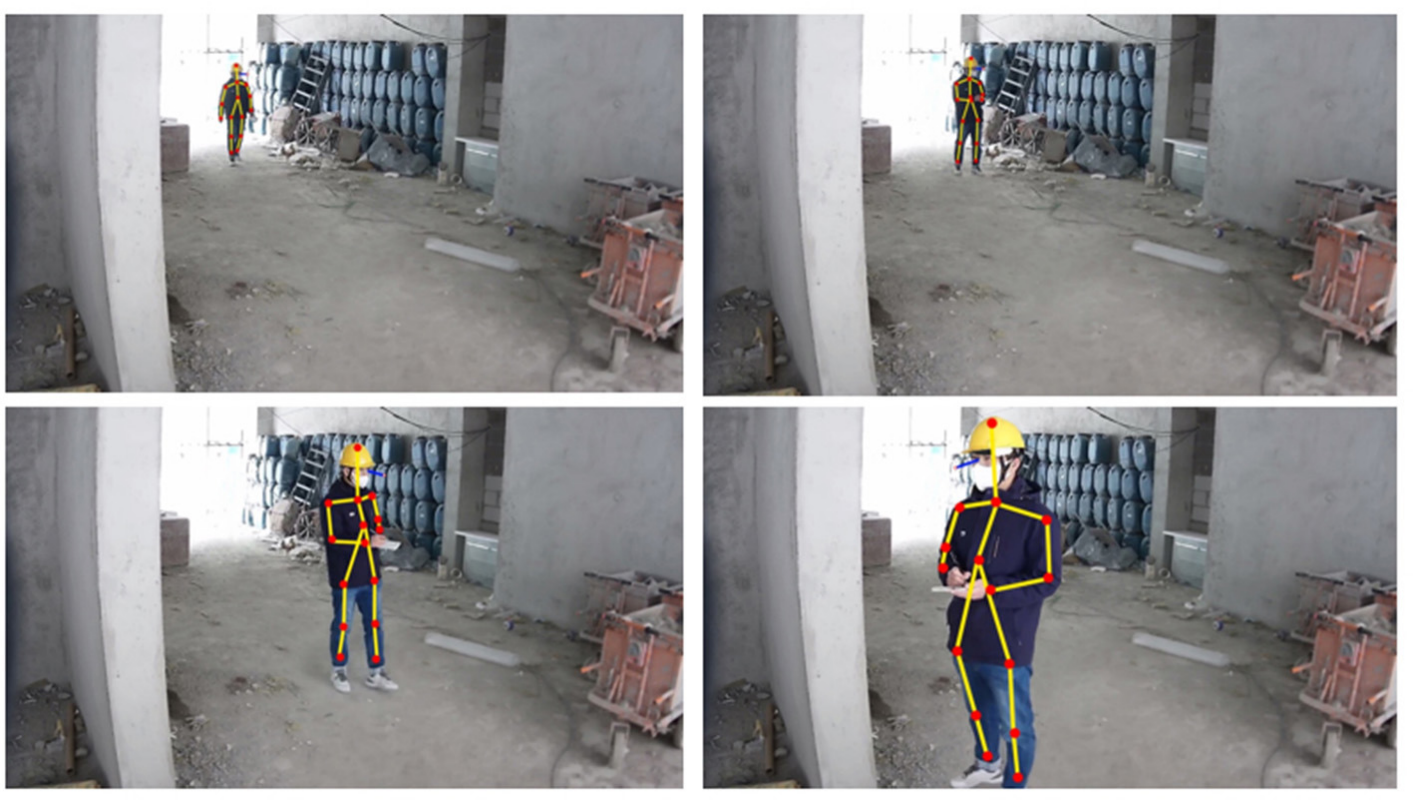
Real-time visualization during the experiment.

The real-time activities of the safety inspector (subject) visualized during the experiment are shown in Figure 11.

Despite the fact that the actual experimental environment needed small number of equipment, the research team increased the number of cameras to 10 and the number of EEGs to 10 and conducted an experiment for 1 h based on the above MCAC framework in order to test the pressure. The performance of the main system is presented in Table 4.

**Table 4:** The main operation results of system performance.

**Indicator**	**Performance**
Number of connections to acquisition server	10 Cameras by wired 10 Portable EEG by wireless
Acquisition server CPU occupancy	40 ~ 65%
Acquisition server memory occupancy	35 ~ 70%
Acquisition server data throughput (AVG)	42,088 kb/s
Control server CPU occupancy	30 ~ 45%
Control server memory occupancy	25 ~ 45%
Control server data throughput (AVG)	6,142 kb/s
Algorithm server CPU occupancy	No special arithmetic operation
Algorithm server memory occupancy	No special arithmetic operation
Algorithm server data throughput (peak value)	No special arithmetic operation
Wireless network delay	<20 ms

## Conclusion

The current trend in psychology and neuroscience is to go out of the lab and use multimodal data to perform a test in a more natural setting. However, this real-time multimodal study, limited by technical limitations, is still rarely reported.

The MCAC framework proposed in this study is a generalized systematic method to support this trend of large-scale application in research. The MCAC framework provides specific technological strategies for the problems that may be encountered during the application of multimodal physiological and neuropsychological techniques in an open VUCA environment. It is therefore a systematic approach to the development of such research studies and a practical technological guide. This article fills a technology gap in the methodology of neuromanagement and automation in VUCA, such as construction scenarios. This is also the main innovation and contribution of this paper.

In the experimental part, we demonstrate the feasibility of this approach by sharing a real test case. Compared with the conventional methods for the same purpose of the experiment, it shows its superiority in improving the efficiency of experiment and data analysis. Also, experiments that are difficult to implement in a complex VUCA environment can now be implemented easily and conveniently.

It is worth noting that the focus of this paper is to find a solution for the dilemma regarding the general application techniques in the fields of neuroscience and psychology, rather than computer architecture. Therefore, we do not discuss systematically the performance and load limits of the computer hardware used in the laboratory, which has nothing to do with the topic of neuromanagement. The objective of this study was to just show that with the support of lightweight, scalable technology architecture, based on common commercial devices like PC instead of large dedicated instruments, it is possible to meet all the requirements of large-scale and complex experiments. This is also a key revelation to the field of psychology and neuroscience.

In the future, we will try to further modify it so that it can be directly applied to the automation of neuromanagement. In addition, the research findings of the above construction experiment will be published.

## Data Availability Statement

The original contributions presented in the study are included in the article/supplementary material, further inquiries can be directed to the corresponding author/s.

## Ethics Statement

Ethical review and approval was not required for the study on human participants in accordance with the local legislation and institutional requirements. The patients/participants provided their written informed consent to participate in this study. Written informed consent was obtained from the individual(s) for the publication of any potentially identifiable images or data included in this article.

## Author Contributions

XQ, YQ, and WD contributed to the conception and design of this study. XQ, YQ, and MW carried out the methodology. YQ, MW, and MC fulfill the technical implementation. YQ and MW performed the practice. XW wrote the original draft of the manuscript. WD managed this manuscript. All authors participated in the revision of the manuscript, read, and approved the submitted version.

## Funding

This work was supported by the National Natural Science Foundation of China (Nos. 71971066 and 72074052), Project of the Ministry of Education of China (No. 18YJA630019), Science and Technology Innovation Action Plan of Shanghai (No. 18411952000), and 2021 Fudan University-Minhang District Health Association Project of Shanghai.

## Conflict of Interest

MW was employed by the company—Shanghai Ineutech Technology Co., Ltd. and MC was employed by the company—China Science IntelliCloud Technology Co., Ltd. The remaining authors declare that the research was conducted in the absence of any commercial or financial relationships that could be construed as a potential conflict of interest.

## Publisher's Note

All claims expressed in this article are solely those of the authors and do not necessarily represent those of their affiliated organizations, or those of the publisher, the editors and the reviewers. Any product that may be evaluated in this article, or claim that may be made by its manufacturer, is not guaranteed or endorsed by the publisher.
